# Dissociative Symptoms in Depressive and Anxiety Disorders: Prevalence, Correlates, and Trajectories During Selective Serotonin Reuptake Inhibitor Treatment

**DOI:** 10.1192/j.eurpsy.2026.10792

**Published:** 2026-07-31

**Authors:** A. Dunalska, A. Jakubczyk, A. Szulc, N. Szejko

**Affiliations:** 1 Psychiatric Clinic of Faculty of Health Sciences, Medical University of Warsaw, Pruszków; 2 2-Department of Psychiatry, Medical University of Warsaw, Warsaw, Poland; 3 Clinic of Psychiatry, Social Psychiatry and Psychotherapy, Hannover Medical School, Hannover, Germany; 4 Department of Bioethics, Medical University of Warsaw, Warsaw, Poland

## Abstract

**Introduction:**

Dissociative symptoms coexist with various psychiatric diagnoses. However, their nature, significance, and impact on treatment as a symptom accompanying depressive and anxiety disorders remain insufficiently researched.

**Objectives:**

The aim was to assess the prevalence and correlations of dissociation in patients with depressive and anxiety disorders before pharmacological treatment and to observe the severity of these symptoms during selective serotonin reuptake inhibitors (SSRI) therapy.

**Methods:**

Adult outpatients (n = 133) with depressive or anxiety disorders were assessed using the Dissociative Experiences Scale- Revised, Somatoform Dissociation Questionnaire-20, Hamilton Depression Rating Scale, and Hamilton Anxiety Rating Scale before pharmacological treatment initiation. In the subgroup who remained on follow-up (n = 100), assessments were repeated after approximately 4 and 8-12 weeks of SSRI therapy. Analyses included correlations, mediation, and nonparametric tests (α = 0.05).

**Results:**

Clinically significant dissociation was found in 12% (DES-R ≥ 71) and 18.8% (SDQ-20 ≥ 35) of patients. Somatoform dissociation correlated moderately with anxiety (r = 0.34, p < 0.001), whereas psychoform dissociation correlated weakly (r = 0.18, p = 0.044). Anxiety fully mediated the relationship between depression and somatoform dissociation. Depression, anxiety, and dissociation scores decreased significantly at follow-up (p < 0.001), with dissociative symptoms showing a smaller and slower reduction than affective and anxiety symptoms. Psychoform dissociation gradually declined, whereas somatoform dissociation reached a plateau after the initial change. Higher baseline levels of psychoform dissociation predicted lower odds of depression remission (OR = 0.86, p = 0.049).

**Conclusions:**

Dissociative symptoms are common and clinically significant in depressive and anxiety disorders. Their prevalence, tendency to persist over time, and association with decreased chance of remission from depressive disorders underscore the need for routine screening for dissociation and the inclusion of psychoeducation and interventions addressing trauma and dissociation in the treatment plan for patients with depressive and anxiety symptoms.

**Disclosure of Interest:**

None Declared

